# The impact of ketamine on pain-related outcomes after thoracotomy: a systematic review with meta-analysis of randomized controlled trials

**DOI:** 10.3389/fmed.2024.1394219

**Published:** 2024-06-11

**Authors:** Aruzhan Zhaksylyk, Yerkin G. Abdildin, Suienish Sultangazin, Aigerim Zhumakanova, Dmitriy Viderman

**Affiliations:** ^1^Department of Surgery, School of Medicine, Nazarbayev University, Astana, Kazakhstan; ^2^Department of Mechanical and Aerospace Engineering, School of Engineering and Digital Sciences, Nazarbayev University, Astana, Kazakhstan; ^3^Department of Anesthesiology, Intensive Care and Pain Medicine, National Research Oncology Center, Astana, Kazakhstan

**Keywords:** thoracic surgery, acute pain, chronic pain, pain management, ketamine, outcomes

## Abstract

**Objective:**

This meta-analysis aims to examine how effective ketamine is in the management of acute and preventing chronic post-thoracotomy pain by synthesizing the available research.

**Method:**

A systematic literature search was conducted across PubMed, Scopus, and Cochrane Library till May 2023. Randomized Controlled Trials (RCT) examining the influence of ketamine on post-thoracotomy pain in adults were included. The intervention group included ketamine plus morphine, while the control group included morphine only. The outcome measures were opioid intake and pain scores at rest and on moving/coughing. Evidence quality was evaluated using the Cochrane Risk of Bias and GRADE assessment.

**Results:**

Nine articles comprising 556 patients were selected for meta-analysis. The intervention group had a significant decrease in pain at rest (Std. Mean Difference (SMD = −0.60 with 95% CI [−0.83, −0.37]) and on movement/cough (SMD = −0.73 [−1.27, −0.18]) in the first postoperative days. Also, the ketamine group had lower opioid consumption (mg) in comparison with controls (SMD = −2.75 [−4.14, −1.36], *p*-value = 0.0001) in postoperative days 1-3. There was no data to assess the long-term effect of ketamine on chronic pain.

**Conclusion:**

This meta-analysis shows that ketamine use can lower acute pain levels and morphine use after thoracotomy. In the future, larger RCTs using standardized methods and assessing both short-term and long-term analgesic effects of ketamine are necessary to deepen the understanding of the issue.

## Introduction

Thoracotomy is a surgical procedure that is performed to access the thoracic cavity organs such as the heart, lungs, thymus, and esophagus (1). It is a common procedure to treat various conditions, including lung cancer, pleural effusions, empyema, aortic dissection, valvular diseases, esophageal cancer, etc. (2). After a thoracotomy, intense and sudden pain is a typical response to various traumatic events. These include rib fractures, the intercostal nerves injury, costovertebral joint dislocation, and resection leading to the irritation of the pleura (3).

Acute pain can have both financial and medical costs, such as longer hospital stays, the requirement for readmission, and patients who are dissatisfied with their medical care (4). According to estimates, a 30-year-old person’s medical expenses for treating chronic pain brought on by acute pain could reach one million USD (5). As a result, treating acute pain with preventative and efficient alleviation techniques can improve clinical outcomes, lower the chance of developing further health issues, conserve healthcare resources, and improve overall quality of life (6).

Despite various anesthesiologic advancements, opioids remain one of the main therapeutic tools. There is a wide distribution of opioid receptors in both central and peripheral nervous systems (7). Sedation, dizziness, constipation, respiratory depression, and tolerance are possible side effects of opioid receptor activation. The development of tolerance, in which the effectiveness of opioids decreases over time requiring higher doses for pain relief, is commonly seen in both acute and chronic use of opioids (8). Patients with opioid tolerance are likely to stay at hospitals longer, and have higher readmission numbers and longer recovery periods (9). Long-term use and higher doses of opioids may cause hyperalgesia, an increased sensitivity to pain associated with opioid intake (8).

Post-thoracotomy pain involves different mechanisms and neural circuits. One of the pathways involved in pain transmission is N-Methyl-D-Aspartate (NMDA) receptor pathway, which is involved in neuropathic pain. NMDA is a receptor of an excitatory neurotransmitter called glutamate that can be released in response to nociception. When a tissue is damaged, it can lead to temporary accumulations of NMDA glutamate receptor postsynaptic depolarizations, which result in an influx of calcium. This activates intracellular signaling pathways, leading to the NMDA receptors phosphorylation, and subsequently causing an extended rise in nociceptive transmission (10). As a result of this sensitization, hyperalgesia and neuropathic pain occur. Additionally, decreased sensitivity of opioid receptors has been linked with NMDA receptor stimulation (11). Lowered sensitivity of opioid receptors, in turn, leads to opioid tolerance. Therefore, NMDA blocking may help with pain management, increasing sensitivity to opioids (12). This has the potential to decrease the dosage of opioid requirements, lowering the incidence of associated side effects.

Ketamine, methadone, memantine, amantadine, and dextromethorphan are a few of the NMDA receptor antagonists that are readily available. While the other NMDA receptor blockers are weaker, ketamine is a potent NMDA antagonist used in emergency medicine and surgical settings. Low-dose ketamine in patient-controlled analgesia for thoracic surgery reduced the use of opioids and improved patients’ respiratory conditions (13). Additionally, a review found that using low-dose ketamine along with morphine after thoracic surgery can lessen postoperative pain and is safer than using morphine alone (14). In another study, however, ketamine failed to improve the effectiveness of epidural anesthesia or prevent pulmonary dysfunction after thoracic surgery (15). Likewise, post-thoracotomy low-dose ketamine did not reduce morphine use or improve pain scores (16). This controversial information demonstrates the ambiguity of data on the efficiency of ketamine for the management of pain following thoracotomy.

This meta-analysis is aimed at examining the effectiveness of ketamine in the management of acute and preventing chronic post-thoracotomy pain by synthesizing the available research. The outcome measures being assessed in this paper are opioid intake, pain alleviation, and pain intensity. The findings of this meta-analysis will aid clinical decision-making and offer useful insights into the usage of ketamine for treating post-thoracotomy pain.

## Methods

The study adhered to the Preferred Reporting Items for Systematic Reviews and Meta-Analyses (PRISMA) guidelines (17). The PRISMA guidelines guided the selection of studies included in this paper, data extraction, and reporting of results. The PRISMA checklist was completed and included in the final manuscript to ensure transparency and completeness of reporting.

### Inclusion criteria

The following eligibility criteria had to be met to include studies:

Patients: 18 years of age or older who have undergone thoracotomy procedure;

Intervention: the use of ketamine solely or in addition to opioids for managing postoperative pain after thoracotomy;

Comparison: Placebo or morphine;

Outcomes: postoperative pain levels and/or the amount of analgesia consumption;

Study design: Randomized, controlled trials (RCTs) with a follow-up period of at least 24 h published in English up to May 2023.

### Exclusion criteria

Non-randomized trials, animal studies, case reports, unpublished observations, abstracts, and study protocols; studies with insufficient data or incomplete reports; studies with a follow-up time of less than 24 h.

#### Data sources and search strategy

PubMed, Scopus, and Cochrane databases were searched for studies. We also searched through the reference lists of the included studies as well as pertinent systematic reviews. For any lacking details on procedures like randomization and blinding, as well as for original data like pain scores, authors were intended to be contacted. The search in each database was done for studies from inception until May 2023. The following keywords and their combinations were used: “esketamine,” “ketamine,” “ketamin,” “ketamine s,” “ketamines,” “pain,” “analgesia”, “opioid,” “pain management,” “postoperative analgesia,” “pain control, “thoracic surgical procedures,” “thoracic,” “surgical, “procedures,” “thoracic,” “surgery,” “thoracic surgery,” “thoracic surgery.”

#### Data extraction and quality assessment

First, a special data extraction form was designed, and filled with the needed data from each eligible study. It included information on details of publication (authors, year and country of study publication), type of study design, sample size, patient’s age, type of thoracotomy procedure performed, the diagnosis of the patients, dose and regimen of analgesia for placebo and study group, and outcomes. The postoperative analgesia outcome factors for the initial 24 h were noted for analysis. These included the VAS score for pain and the total amount of postoperative opioids. The visual analog score (VAS, 0–10 cm, where 0 = no pain, 10 = maximum pain) was employed to analyze the pain outcome quantitatively. Verbal rating pain scores (VRS, 0–100 mm), numerical rating scores, and other comparable values were converted into VAS pain scores for analysis. The mean VAS for each therapy group was estimated for each study within 24 h of surgery using all available observations.

All identified studies’ titles and abstracts were separately reviewed by two authors to identify their eligibility status. Then, two authors independently retrieved and read the full-texts of potentially eligible publications. All disagreements were settled through consensus-building or discussion. The authors and institutions of the studies were hidden from the reviewers.

The quality of each study was evaluated using the GRADE method (18) and the Cochrane Risk of Bias tool 2 (19). The risk of bias was evaluated for each of the following aspects of the Cochrane Collaboration’s tool 2: bias arising from the randomization process, deviations from intended interventions, missing outcome data, measurement of the outcome, selection of the reported results, and the overall risk of bias. The overall risk of bias was assessed as low, high, or having some concerns. The GRADE method was used to grade the strength of the evidence supporting each outcome. Based on the likelihood of bias, inconsistent findings, indirectness, and imprecision, the quality of the evidence was assessed as high, moderate, low, or very low. Two reviewers separately assessed the potential for bias in each eligible study, and any discrepancies and mismatches were resolved via discussion.

We extracted and entered descriptive data in a table. “Review Manager (RevMan) [Computer program]. Version 5.4. The Cochrane Collaboration, 2020” was used to conduct data analysis. I^2^ statistic was used to evaluate statistical heterogeneity. Whenever needed, mathematical methods for estimating sample mean and standard deviation were applied (20, 21).

## Results

**Figure 1:**
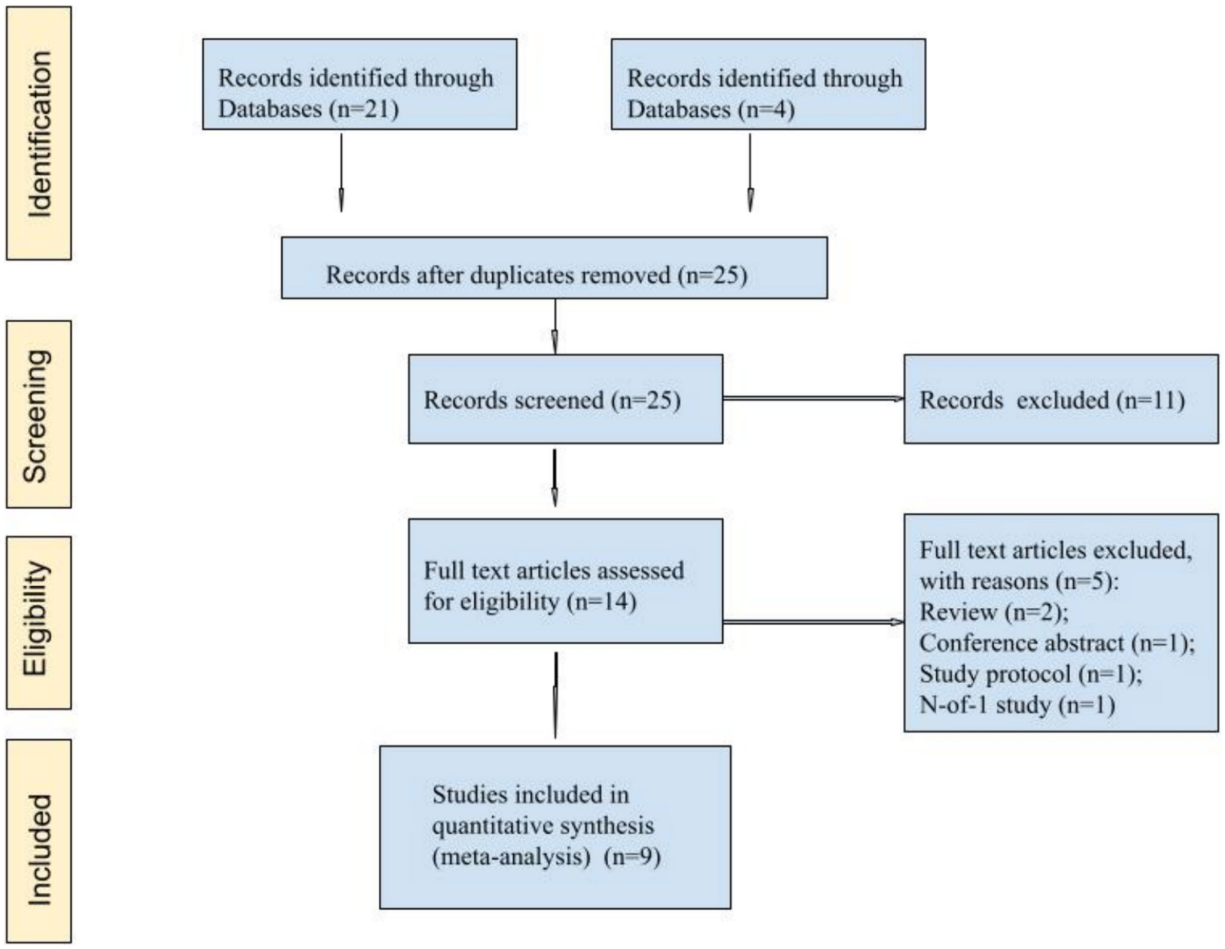
PRISMA. The flow of the study selection process.

Overall, nine articles were found that matched the search criteria (Figure 1). Nine articles (13, 22–29) comprising 556 patients were selected for meta-analysis (Table 1).

**Table 1:** Study characteristics.

Author, year, country, study design	Study goals	Age (ketamine/control)	Groups, *N* of patients: total (ketamine/control)	Surgery	Dose and regimen	Conclusions
Chumbley, 2019, UK, RCT	The effect of intraoperative ketamine use on pain	61 (26–77) 68 (48–87)	70 Ketamine (35) Placebo (35)	Thoracotomy for primary carcinoma, metastatic disease, benign tumor	Continuously for 96 h Ketamine group: 0.1 mg kg − 1 h – 1 Placebo group: Normal saline	Reduced pain and opioid use in the first 48 h but no difference in persistent long-term pain
Fiorelli, 2015, Italy, RCT	The effect of preemptive ketamine on pain	59.5 ± 15.3 58.6 ± 17.4	75 ITT Ketamine (38) Placebo (37)	Thoracotomy for non-small-cell lung cancer	Ketamine group: 1 mg/kg ketamine Placebo group: Normal saline	Lower pain scores and morphine use in the ketamine group
Tena, 2014, Spain, RCT	The effect of IV or epidural ketamine+ TEA	Group S: 66.5 ± 9.9 Group Kiv: 62.9 ± 9.8 Group Kep:63.4 ± 11.9	104 Ketamine IV (36) Ketamine epidural (33) Placebo (35)	Thoracotomy (pulmonary resection)	Kiv: IV racemic ketamine 0.5 mg/kg preincisional +0.25 mg/kg/h for 48 h Kep: epidural racemic ketamine 0.5 mg/kg preincisional +0.25 mg/kg/h for 48 h S: saline	Lower pain scores at 24 and 72 h (ketamine vs. control)
Chazan, 2005, Israel, RCT	The effect of ketamine+morphine on pain	60 ± 16 57 ± 18	46 Morphine + ketamine (24) Morphine alone (22)	Minimally invasive direct coronary artery bypass, off-pump coronary artery bypass, or thoracotomy	Morphine + ketamine: 1 mg morphine +5 mg ketamine/bolus Morphine alone: 2 mg/bolus	Lower opioid consumption and better pain relief in the ketamine group
Dualé, 2009, France, RCT	The effect of intraoperative ketamine on persistent neuropathic pain	61.9 ± 8.3 58.5 ± 8.5	86 Ketamine (42) Placebo (44)	Thoracotomy	Ketamine: 500 mg in 500 mL, 1 mg kg^−1^ at induction, during surgery, for 24 h Placebo: normal saline	Lower morphine use at 24 h and lower pain scores at 48 h in the ketamine group. No difference in persistent pain.
Nesher, 2008, Israel, RCT	The effect of sub-anesthetic dose of ketamine with morphine on pain, drowsiness, hemodynamic and respiratory outcomes	60 ± 15 59 ± 16	57 Morphine+ketamine (28) Morphine only (29)	Thoracotomy for MIDCAB, lung tumor resection, or median sternotomy for OPCAB	MO: 1.5 mg MO bolus MK: 1.0 mg MO + 5 mg ketamine/bolus	Lower morphine use and pain scores for 72 h in the ketamine group
Nesher, 2009, Israel, RCT	The effect of sub-anesthetic dose of ketamine with 2/3 morphine dose on pain	61 ± 11 58 ± 12	41 Morphine+ketamine (21) Morphine only (20)	Thoracotomy for minimally invasive direct coronary artery bypass or for lung tumor resection	MK: 1.0 mg MO + 5-mg ketamine bolus MO-only: 1.5 mg MO + saline	Lower morphine use and side effects in the ketamine group
Pehlivan, 2019, Turkey, RCT	The effect of perioperative low-dose ketamine on acute and chronic pain	50.15 ± 10.23 45.5 ± 10.99	40 Ketamine (20) Saline (20)	Lung resection, pneumonectomy, biopsy, decortication, wedgeresection, bulla excision, massexcision, cystexcision	Ketamine: ketamine 0.5 mg.kg-1 bolus, 2 mcg.kg-1.dk-1 infusion for 24 h before the incision Placebo: normal saline	Lower acute pain and morphine use in ketamine group. No difference in chronic pain.
Michelet, 2007, France, RCT	The effect of ketamine + morphine PCA on morphine consumption and postoperative respiratory disorders	64 (42–77) 63 (42–76)	50 Morpine+ketamine (25) Morphine only (25)	Lobectomy by posterolateral thoracotomy incision	Morpine+ketamine: morphine with ketamine 1 mg ml^−1^ for each. Morphine only: IV morphine 1 mg ml^−1^	Lower morphine consumption in the ketamine group

ITT, intention-to-treat; IV, intravenous; TEA, thoracic epidural analgesia.

### VAS pain intensity score at rest during the first four postoperative days (0–10)

**Figure 2:**
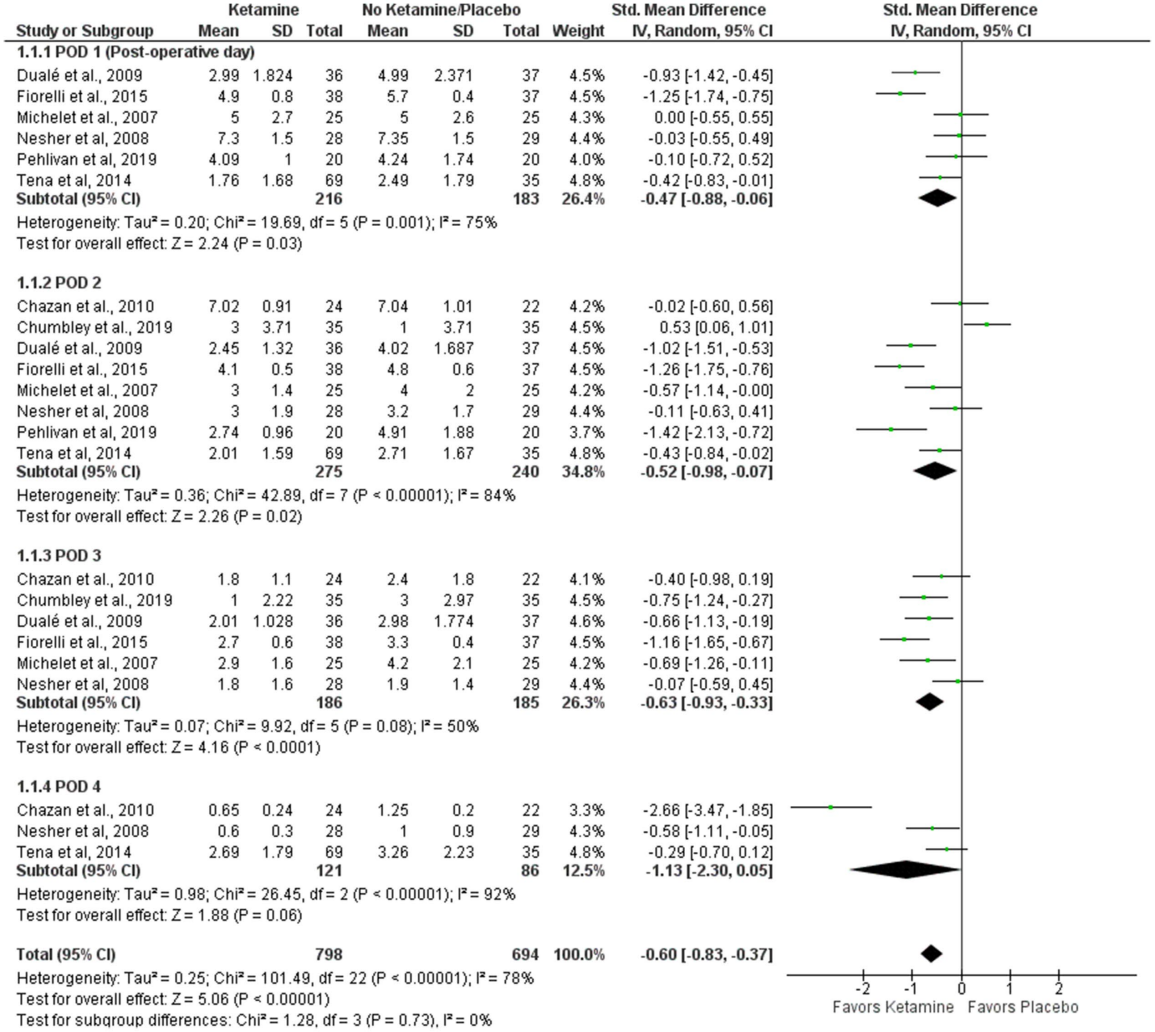
Pain intensity at rest.

VAS scores for pain intensity at rest are presented in a forest plot (Figure 2). The analyzed studies used two types of control-intervention division: some papers distributed their patients into Ketamine-Morphine and Only Morphine groups, such as Chazan et al. (25) and Michelet et al. (13), while others had Ketamine and Placebo groups like Chumbley et al. (22) and Fiorelli et al. (23). The experimental group included “Ketamine-Morphine” and “Ketamine,” while the control group included “Morphine only” and “Placebo” groups.

The patients were asked to rate the amount of pain intensity on a 0–10 scale or 0–100 scale in some cases, such as Duale et al. (26) and Michelet et al. (13). All scores were rescaled to 0–10 (see Tables 2, 3).

**Table 2:** Cochrane risk of bias tool 2.

Study reference	D1	D2	D3	D4	D5	Overall		
Chumbley et al. (22)	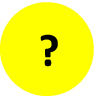	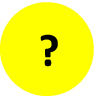	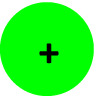	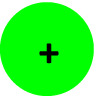	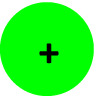	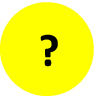	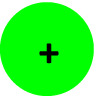	Low risk
Fiorelli et al. (23)	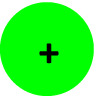	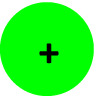	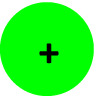	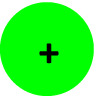	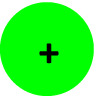	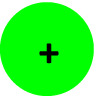	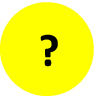	Some concerns
Tena et al. (24)	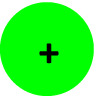	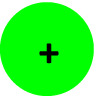	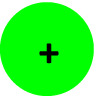	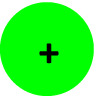	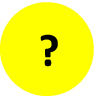	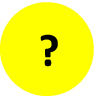	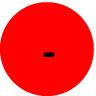	High risk
Chazan et al. (25)	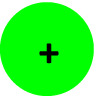	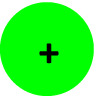	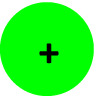	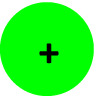	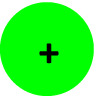	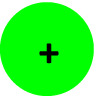		Bias arising from:
Dualé et al. (26)	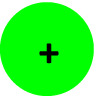	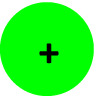	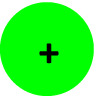	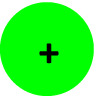	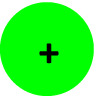	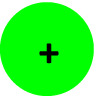	D1	Randomization process
Nesher et al. (27)	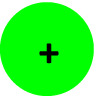	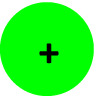	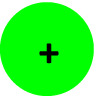	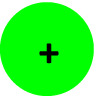	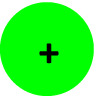	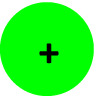	D2	Deviations from the intended interventions
Nesher et al. (28)	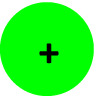	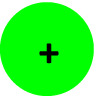	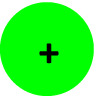	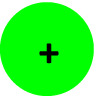	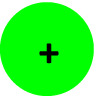	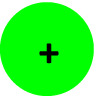	D3	Missing outcome data
Pehlivan et al. (29)	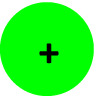	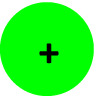	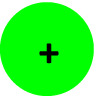	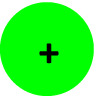	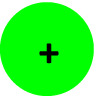	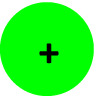	D4	Measurement of the outcome
Michelet et al. (13)	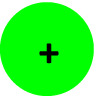	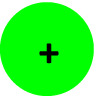	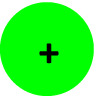	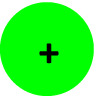	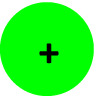	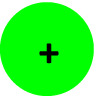	D5	Selection of the reported result

**Table 3:** Summary of findings.

	N of studies	Design	*N* of patients		Effect	Overall quality
Outcome			Intervention	Control	Standardized mean difference [95% CI]	
Cumulative pain intensity at rest	8	RCT	798	694	−0.60 [−0.83, −0.37]	Moderate ⨁⨁⨁⊖
Cumulative pain intensity on coughing/movement	4	RCT	160	127	−0.73 [−1.27, −0.18]	Moderate ⨁⨁⨁⊖
Opioid consumption	6	RCT	332	326	−2.75 [−4.14, −1.36]	Moderate ⨁⨁⨁⊖

CI, confidence interval; N, number; RCT, randomized controlled trial.

Overall, the model favors the intervention group over the placebo group (Std. Mean Difference with 95% CI: −0.60 [−0.83, −0.37]). This result is not sensitive to the exclusion of individual studies. The heterogeneity of the model is considerable (I^2^ = 78%). The weights were distributed evenly in the first and third PODs, while POD 4 weighted 12%, and POD 2 had 35%.

### VAS pain intensity score while moving or coughing during the first four postoperative days (0–10)

**Figure 3:**
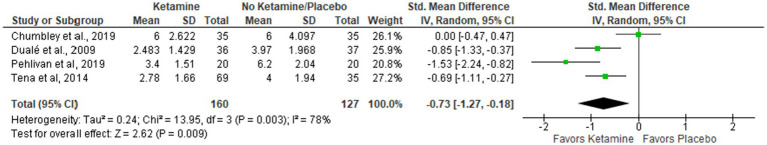
Pain intensity on coughing/movement.

The data for four PODs were merged into one subgroup due to a lack of data for dividing it into categories. Overall, the results were similar to the previous one, but here three papers favored the Ketamine group, while Chumbley et al. (22) were neutral. The model supports the ketamine group (Std. Mean Difference with 95% CI: −0.73 [−1.27, −0.18]), but the result is sensitive to the exclusion of either of these two studies: Dualé et al. (26) or Tena et al. (24) (see Figure 3).

### Cumulative opioid consumption during the first 3 PODs (mg of morphine)

**Figure 4:**
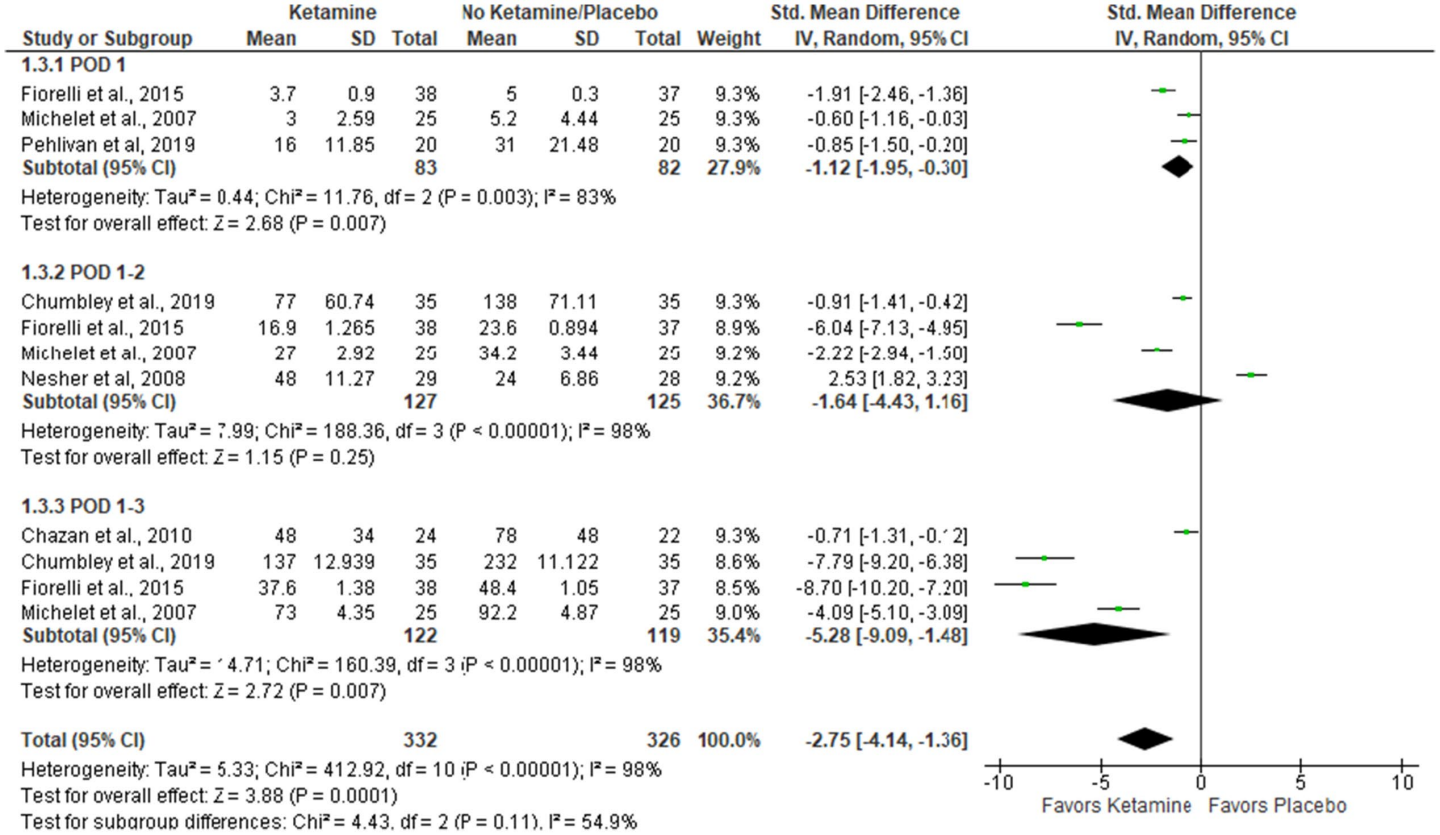
Cumulative opioid consumption.

The overall effect of the model supports the ketamine group compared to placebo (Std. Mean Difference with 95% CI: −2.75 [−4.14, −1.36], *p*-value = 0.0001). In subgroup analysis, the model favors the ketamine group on POD 1 and POD 1–3, but these results are sensitive to the exclusion of studies by Pehlivan et al. (29) and Michelet et al. (13), respectively (see Figure 4).

## Discussion

The management of pain following thoracotomy is crucial for the respiratory system. The pain may restrict breathing and cause the reflective contraction of expiratory muscles, consequently leading to diaphragmatic dysfunction, decreased functional residual capacity, atelectasis, and hypoxemia. Moreover, patients are often extubated early to lower the risk of pulmonary barotrauma and respiratory infections. However, deep breathing necessitates the stretching of an incision, and when the pain is inadequately controlled, patients avoid this stretching via contraction of expiratory muscles. This lack of deep inspiration before the exhalation leads to ineffective coughing and the retention of secretions, which may lead to atelectasis. This highlights the need for adequate pain management to prevent reintubation due to inadequate pulmonary hygiene (30).

Acute pain may also lead to the development of chronic post-thoracotomy pain syndrome (PTPS) (31). PTPS is an incision-area pain, whereby patients experience tenderness and a burning feeling that is accompanied by persistent discomfort. PTPS is frequently associated with central sensitization and is thought to be caused by a combination of peripheral and central nerve injury. The release of mediators following tissue damage activates the nociceptive receptors. These mediators include prostanoids as prostaglandins and leukotrienes, histamine, kinins, and substance P (32). Increased neuron excitability caused by repeated exposure to pain stimuli results in chronic pain that lasts longer than the initial injury or inflammation (3).

Post-thoracotomy pain remains a serious issue despite improvements in surgical methods and pain management procedures, and the incidence of post-thoracotomy pain syndrome can range from 30 to 50% (33). Patients may have moderate to severe pain immediately following surgery, and this discomfort may last during the patient’s hospital stay and the first month following the operation. Furthermore, there might be residual chronic pain that continues for several months to years which can have a major influence on their day-to-day activities in the long run. Studies show that about half of patients have pain that lasts up to a year after surgery, and about one-third reports pain lasting for up to four years (3).

In this meta-analysis, ketamine groups consistently showed lower levels of pain at rest compared to controls during the first postoperative days. Pain levels on movement or cough were also lower in the experimental group. Moreover, the overall opioid requirement was lower compared to control over the first three postoperative days. There was no data to assess the long-term effects of ketamine on chronic pain.

Our findings align with research conducted by Moyse et al. (34), which similarly reported ketamine’s positive effects on acute pain following thoracotomy. The researchers also emphasized the lack of evidence supporting its efficacy in reducing chronic post-thoracotomy pain. A systematic review and meta-analysis by McNicol et al. (35) suggested limited support for ketamine’s role in preventing chronic pain outcomes, despite offering short-term relief from persistent post-surgical pain. Sun et al. (36) provided insights into the potential benefits of intravenous ketamine in reducing the incidence of chronic postsurgical pain (CPSP) at three to six months postoperatively. Their findings suggest that perioperative IV ketamine may have a positive effect on reducing CPSP compared to placebo.

Despite these favorable results of ketamine use in improving postoperative pain management, there are several limitations of the current study. First, the heterogeneity of study designs, surgeries included in studies, and VAS score measuring times may contribute to study results. Also, the pain score scales for pain at rest, movement, and coughing may differ between the studies. Studies differ in the way that they report pain estimates at different time points. It may introduce temporal heterogeneity. It might also introduce selective reporting bias into the study. In order to increase the comparability and overall quality of studies, future research should aim at more standardized methods like using the same standardized pain scales as well as measuring pain scores at the same standardized time points. In addition, a smaller sample size may overestimate the effects of ketamine, thus in the future, there should be more RCT with a bigger sample size. Moreover, the available studies may lack comprehensive and prolonged studies on ketamine safety as well as efficacy beyond a few months or years, so it is not fully possible to assess the ketamine effect duration and side effects on post-thoracotomy patients over extended periods. Therefore, while interpreting this current evidence, this limitation should be considered with caution. To gain a better understanding of the ketamine impact on long-term post-thoracotomy pain management, the study should focus on long-term safety with follow-up.

Our meta-analysis shows that ketamine has a potential to be used in clinical practice to improve pain control, thus it can possibly be added to pain management protocols. Moreover, as it can decrease the opioid dose required, its use can improve opioid tolerance and addiction problems, as well as opioid side effects in patients after thoracotomy.

However, ketamine should be explored very carefully before applying it for post-thoracotomy pain control in clinical practice. Future research should focus on aspects like dose optimization for patients. The optimal dose can depend on different factors like patient surgery type, demographics of the patient, or pain intensity. Also, to gain a better understanding of pain control achieved, future studies can be focused on patient-reported outcomes. This can include patient-reported quality of life, satisfaction levels with pain control, and physical functioning.

Furthermore, although this research compared ketamine with opioid use for pain management with opioid only strategy, future studies may investigate other pain management techniques including regional anesthesia techniques, such epidural, paravertebral block, erector spinae plane block, which have been shown their efficacy in different surgeries (37–39). Also, drugs alpha-adrenergic agonists (dexmedetomidine), gabapentinoids, acetaminophen could be used to compare and contrast their effect on opioid consumption. These comparative studies would give an opportunity to widely assess the efficacy of each pain management modality.

## Conclusion

In conclusion, this meta-analysis shows that ketamine can lower pain levels after thoracotomy. Combining the results of several randomized controlled trials, this study shows that ketamine has the potential to be used in postoperative analgesia and decrease opioid tolerance. In the future, more RCT involving larger sample sizes and more standardized methods of measurements are necessary to add to the body of knowledge on the topic.

## Data availability statement

The original contributions presented in the study are included in the article/supplementary material, further inquiries can be directed to the corresponding author.

## Author contributions

ArZ: Writing – original draft, Writing – review & editing. YA: Writing – original draft, Writing – review & editing, Data curation, Formal analysis. SS: Data curation, Writing – review & editing, Formal analysis. AiZ: Writing – review & editing. DV: Conceptualization, Funding acquisition, Methodology, Project administration, Supervision, Writing – original draft, Writing – review & editing.

## References

[1] Hachenberg T Loop T. Postthoracotomy complications. In: Cohen’s comprehensive thoracic anesthesia. Elsevier (2022). 376–91.

[2] Chang B Tucker WD Burns B. Thoracotomy. StatPearls, Treasure Island (FL): StatPearls Publishing (2024). 32491532

[3] Ochroch EA Gottschalk A. Impact of acute pain and its Management for Thoracic Surgical Patients. Thorac Surg Clin. (2005) 15:105–21. doi: 10.1016/j.thorsurg.2004.08.004 15707349

[4] Joshi GP Ogunnaike BO. Consequences of inadequate postoperative pain relief and chronic persistent postoperative pain. Anesthesiol Clin North Am. (2005) 23:21–36. doi: 10.1016/j.atc.2004.11.013, PMID: 15763409

[5] Cousins M Power I Smith G. 1996 Labat lecture: Pain—A persistent problem. Reg Anesth Pain Med. (2000) 25:6–21. doi: 10.1016/S1098-7339(00)80005-X, PMID: 10660235

[6] Apfelbaum JL Chen C Mehta SS Gan ATJ. Postoperative pain experience: results from a National Survey Suggest Postoperative Pain Continues to be undermanaged. Anesth Analg. (2003) 97:534–40. doi: 10.1213/01.ANE.0000068822.10113.9E, PMID: 12873949

[7] Pathan H Williams J. Basic opioid pharmacology: an update. Br J Pain. (2012) 6:11–6. doi: 10.1177/2049463712438493, PMID: 26516461 PMC4590096

[8] Benyamin R Trescot AM Datta S Buenaventura R Adlaka R Sehgal N . Opioid complications and side effects. Pain Physician. (2008) 11:S105–20. doi: 10.36076/ppj.2008/11/S105, PMID: 18443635

[9] Gulur P Williams L Chaudhary S Koury K Jaff M. Opioid tolerance—a predictor of increased length of stay and higher readmission rates. Pain Physician. (2014) 4:E503–7. doi: 10.36076/ppj.2014/17/E503 25054400

[10] Zhou H-Y Chen S-R Pan H-L. Targeting N -methyl- D -aspartate receptors for treatment of neuropathic pain. Expert Rev Clin Pharmacol. (2011) 4:379–88. doi: 10.1586/ecp.11.17, PMID: 21686074 PMC3113704

[11] Petrenko AB Yamakura T Baba H Shimoji K. The role of N-methyl-D-aspartate (NMDA) receptors in pain: a review. Anesth Analg. (2003) 97:1108–16. doi: 10.1213/01.ANE.0000081061.12235.55, PMID: 14500166

[12] DuPen A Shen D Ersek M. Mechanisms of opioid-induced tolerance and hyperalgesia. Pain Manag Nurs. (2007) 8:113–21. doi: 10.1016/j.pmn.2007.02.004 17723928

[13] Michelet P Guervilly C Hélaine A Avaro JP Blayac D Gaillat F . Adding ketamine to morphine for patient-controlled analgesia after thoracic surgery: influence on morphine consumption, respiratory function, and nocturnal desaturation. Br J Anaesth. (2007) 99:396–403. doi: 10.1093/bja/aem168, PMID: 17576969

[14] Mathews TJ Churchhouse AMD Housden T Dunning J. Does adding ketamine to morphine patient-controlled analgesia safely improve post-thoracotomy pain? Interact Cardiovasc Thorac Surg. (2012) 14:194–9. doi: 10.1093/icvts/ivr081, PMID: 22159259 PMC3279980

[15] Joseph C Gaillat F Duponq R Lieven R Baumstarck K Thomas P . Is there any benefit to adding intravenous ketamine to patient-controlled epidural analgesia after thoracic surgery? A randomized double-blind study. Eur J Cardiothorac Surg. (2012) 42:e58–65. doi: 10.1093/ejcts/ezs398, PMID: 22790008

[16] Yazigi A Abou-Zeid H Srouji T Madi-Jebara S Haddad F Jabbour K. The effect of low-dose intravenous ketamine on continuous intercostal analgesia following thoracotomy. Ann Card Anaesth. (2012) 15:32–8. doi: 10.4103/0971-9784.91479, PMID: 22234019

[17] Page MJ McKenzie JE Bossuyt PM Boutron I Hoffmann TC Mulrow CD . The PRISMA 2020 statement: an updated guideline for reporting systematic reviews. BMJ. (2021) 372:n71. doi: 10.1136/bmj.n71, PMID: 33782057 PMC8005924

[18] Guyatt GH Oxman AD Schünemann HJ Tugwell P Knottnerus A. GRADE guidelines: a new series of articles in the journal of clinical epidemiology. J Clin Epidemiol. (2011) 64:380–2. doi: 10.1016/j.jclinepi.2010.09.011, PMID: 21185693

[19] Sterne JAC Savović J Page MJ Elbers RG Blencowe NS Boutron I . RoB 2: a revised tool for assessing risk of bias in randomised trials. BMJ. (2019) 366:l4898. doi: 10.1136/bmj.l4898 31462531

[20] Wan X Wang W Liu J Tong T. Estimating the sample mean and standard deviation from the sample size, median, range and/or interquartile range. BMC Med Res Methodol. (2014) 14:135. doi: 10.1186/1471-2288-14-135, PMID: 25524443 PMC4383202

[21] Luo D Wan X Liu J Tong T. Optimally estimating the sample mean from the sample size, median, mid-range, and/or mid-quartile range. Stat Methods Med Res. (2018) 27:1785–805. doi: 10.1177/0962280216669183, PMID: 27683581

[22] Chumbley GM Thompson L Swatman JE Urch C. Ketamine infusion for 96 hr after thoracotomy: effects on acute and persistent pain. Eur J Pain. (2019) 23:985–93. doi: 10.1002/ejp.1366, PMID: 30719817

[23] Fiorelli A Mazzella A Passavanti B Sansone P Chiodini P Iannotti M . Is pre-emptive administration of ketamine a significant adjunction to intravenous morphine analgesia for controlling postoperative pain? A randomized, double-blind, placebo-controlled clinical trial. Interact Cardiovasc Thorac Surg. (2015) 21:284–90. doi: 10.1093/icvts/ivv154, PMID: 26071592

[24] Tena B Gomar C Rios J. Perioperative epidural or intravenous ketamine does not improve the effectiveness of thoracic epidural analgesia for acute and chronic pain after thoracotomy. Clin J Pain. (2014) 30:490–500. doi: 10.1097/AJP.0000000000000005 24281290

[25] Chazan S Buda I Nesher N Paz J Weinbroum AA. Low-dose ketamine via intravenous patient-controlled analgesia device after various transthoracic procedures improves analgesia and patient and family satisfaction. Pain Manag Nurs. (2010) 11:169–76. doi: 10.1016/j.pmn.2009.06.003, PMID: 20728066

[26] Dualé C Sibaud F Guastella V Vallet L Gimbert Y-A Taheri H . Perioperative ketamine does not prevent chronic pain after thoracotomy. Eur J Pain. (2009) 13:497–505. doi: 10.1016/j.ejpain.2008.06.013 18783971

[27] Nesher N Serovian I Marouani N Chazan S Weinbroum AA. Ketamine spares morphine consumption after transthoracic lung and heart surgery without adverse hemodynamic effects. Pharmacol Res. (2008) 58:38–44. doi: 10.1016/j.phrs.2008.06.003, PMID: 18602474

[28] Nesher N Ekstein MP Paz Y Marouani N Chazan S Weinbroum AA. Morphine with adjuvant ketamine vs higher dose of morphine alone for immediate postthoracotomy analgesia. Chest. (2009) 136:245–52. doi: 10.1378/chest.08-0246 18753471

[29] Pehlivan S Ulgey A Bayram A Biçer C Oğuzkaya F Boyacı A. The effect of low dose ketamine infusion on postoperative acute and chronic pain after thoracotomy. Dicle Tıp Dergisi. (2019) 46:677–84. doi: 10.5798/dicletip.661256

[30] Gerner P. Postthoracotomy pain management problems. Anesthesiol Clin. (2008) 26:355–67. doi: 10.1016/j.anclin.2008.01.007, PMID: 18456219 PMC2453516

[31] Perkins FM Kehlet H. Chronic pain as an outcome of surgery. Anesthesiology. (2000) 93:1123–33. doi: 10.1097/00000542-200010000-00038 11020770

[32] Kelly DJ Ahmad M Brull SJ. Preemptive analgesia I: physiological pathways and pharmacological modalities. Can J Anesth/J Can Anesth. (2001) 48:1000–10. doi: 10.1007/BF03016591, PMID: 11698320

[33] Khelemsky Y Noto CJ. Preventing post-thoracotomy pain syndrome. Mount Sinai J Med. (2012) 79:133–9. doi: 10.1002/msj.21286, PMID: 22238046

[34] Moyse DW Kaye AD Diaz JH Qadri MY Lindsay D Pyati S. Perioperative ketamine Administration for Thoracotomy Pain. Pain Physician. (2017) 20:173–84. PMID: 28339431

[35] McNicol ED Schumann R Haroutounian S. A systematic review and meta-analysis of ketamine for the prevention of persistent post-surgical pain. Acta Anaesthesiol Scand. (2014) 58:1199–213. doi: 10.1111/aas.12377, PMID: 25060512

[36] Sun W Zhou Y Wang J Fu Y Fan J Cui Y . Effects of ketamine on chronic postsurgical pain in patients undergoing surgery: a systematic review and Meta-analysis. Pain Physician. (2023) 26:E111–22. PMID: 37192226

[37] Viderman D Aubakirova M Umbetzhanov Y Kulkaeva G Shalekenov SB Abdildin YG. Ultrasound-guided erector spinae plane block in thoracolumbar spinal surgery: a systematic review and meta-analysis. Front Med. (2022) 9:932101. doi: 10.3389/fmed.2022.932101, PMID: 35860731 PMC9289466

[38] Urits I Charipova K Gress K Laughlin P Orhurhu V Kaye AD . Expanding role of the erector spinae plane block for postoperative and chronic pain management. Curr Pain Headache Rep. (2019) 23:1–6. doi: 10.1007/s11916-019-0812-y 31372769

[39] Viderman D Dautova A Sarria-Santamera A. Erector spinae plane block in acute interventional pain management: a systematic review. Scand J Pain. (2021) 21:671–9. doi: 10.1515/sjpain-2020-0171, PMID: 33984888

